# The sense behind retroviral anti-sense transcription

**DOI:** 10.1186/s12985-016-0667-3

**Published:** 2017-01-14

**Authors:** Mamneet Manghera, Alycia Magnusson, Renée N. Douville

**Affiliations:** 1 Department of Immunology, University of Manitoba, Winnipeg, MB Canada; 2 Department of Biology, The University of Winnipeg, Winnipeg, MB Canada

**Keywords:** Viral genomes, Retrovirus, Human endogenous retrovirus-K, Antisense transcription, Long-terminal repeat (LTR), Transcription factors, Conserved protein domains

## Abstract

Retroviruses are known to rely extensively on the expression of viral proteins from the sense proviral genomic strand. Yet, the production of regulatory retroviral proteins from antisense-encoded viral genes is gaining research attention, due to their clinical significance. This report will discuss what is known about antisense transcription in *Retroviridae,* and provide new information about antisense transcriptional regulation through a comparison of Human Immunodeficiency Virus (HIV), Human T-cell Lymphotrophic Virus (HTLV-1) and endogenous retrovirus-K (ERVK) long terminal repeats (LTRs). We will attempt to demonstrate that the potential for antisense transcription is more widespread within retroviruses than has been previously appreciated, with this feature being the rule, rather than the exception.

## Main text

Retroviruses share a common genomic organization in which the 5′ long terminal repeat (LTR) is followed by the *gag*, *pro*, *pol* and *env* genes, and terminates with the 3′ LTR. Accessory genes are encoded in ways unique to each viral species. The majority of viral protein products stem from the translation of sense-strand RNA transcripts. Until recently, retroviral antisense transcription has been largely overlooked as a source of viral RNA and proteins. However, there is accumulating evidence of antisense transcription in numerous exogenous retroviral genera, including lentiviruses, deltaretroviruses, gammaretroviruses and betaretroviruses. Thus, the expression of antisense proteins may be a broad phenomenon occurring across *Retroviridae*, suggesting that antisense encoded genes are an integral part of the viral genome. This report contributes to our understanding of antisense transcription by characterizing exogenous and endogenous retroviral 3ʹ (antisense) promoters. Our results highlight that antisense transcription may be more widespread than previously appreciated, with endogenous retroviruses (ERVs) incapable of antisense transcription being the exception, rather than the rule.

## Antisense transcription among exogenous retroviruses

Antisense transcription is much better understood in exogenous retroviruses, as compared to their endogenous counterparts. Human Immunodeficiency Virus (HIV) and Human T-cell Lymphotrophic Virus (HTLV) are the characterized retroviruses exhibiting this phenomenon. The proteins encoded by their antisense strands serve important functions, including control of viral sense transcription, as well as viral latency, pathology, and spread [1–4].

### HIV antisense transcription

The ability of HIV-1 to encode an antisense protein was suspected as early as 1988, when a conserved ORF, later named *asp-1*, was identified in the region complementary to the *env* gene in many HIV-1 strains [5]. Since then, many studies have confirmed the expression of ASP-1 RNA and protein *in vitro* in various HIV-expressing cell types, including monocyte-derived macrophages, dendritic cells, and T cells [6–10]. Antibodies recognizing an antisense protein derived from the *env* gene have also been identified in HIV^+^ patients [11]*.* Recently, ASP-1 has been shown to induce autophagy, which may explain its low abundance in HIV-1 infected cells and the inherent difficulty in detecting this protein [12]. ASP-1 has been postulated to utilize this pathway to enhance HIV-1 replication, as wild-type, but not mutated forms of this antisense protein, resulted in optimal viral replication through stimulation of autophagy [12]. In contrast, antisense transcript variants corresponding to proviral *asp* have been shown to inhibit HIV-1 replication *in vitro* in acutely and chronically infected cell lines, as well as in HIV-1 infected human PBMCs [8]. This suggests that HIV-1 antisense transcription may have a critical role in establishing viral latency. Moreover, ASP-1 can form stable cytosolic aggregates, which may sequester essential cellular proteins, and thus modulate the function of cellular pathways in HIV-1 infected cells [12]. Nonetheless, the precise functions of viral antisense RNA and protein during HIV-1 infection *in vivo* remain to be clearly elucidated.

The 3′ LTR promoter is crucial for driving antisense transcription in HIV-1. It has been experimentally demonstrated that HIV-1 antisense transcription initiates at multiple positions in the U3 region of the 3′ LTR, as well as at other downstream regions within the antisense strand [8]. Other studies have reported that HIV-1 antisense transcription is initiated in the U5 region of the 3′ LTR [7, 13]. The multiplicity of transcription initiation sites may be a consequence of the lack of a TATA box in the HIV-1 3′ LTR [6], in which case transcription is initiated through alternative core promoter elements called initiator (INR) motifs (YYANWYY) [7, 14]. The presence of these multiple INR motifs serves to explain the variability observed in transcription initiation sites reported by different studies.

**Fig. 1:**
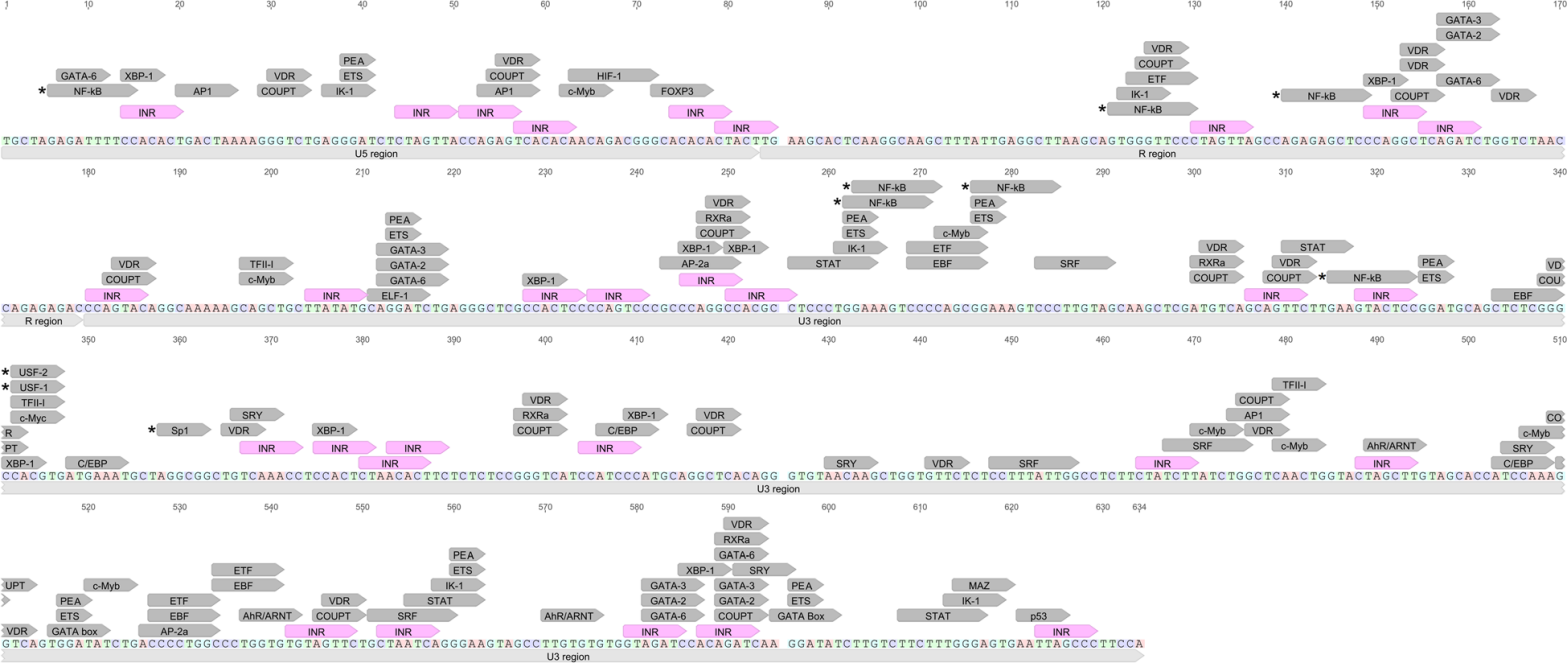
*In silico* examination of transcription factor binding sites and response elements within the representative HIV-1 3′ LTR using ALGGEN-PROMO software [73]. The prototypic HIV-1 3′ LTR sequence used was obtained from GenBank, accession number K03455 (HXB2 strain). The known binding sites for NF-κB, Sp1, USF-1, and USF-2 transcription factors in the HIV-1 3′ LTR are flagged with an asterisk. Initiator motifs (INRs) are indicated in pink. Sequence annotations were performed using Geneious software [68]

In comparison to that of its sense transcription, the regulation of HIV-1 antisense transcription is not well understood. Nonetheless, several host transcription factors have been shown to play key roles in inducing transcription from the antisense strand of HIV-1, including Specificity Protein-1 (Sp1) [15, 16] Upstream Stimulating Factor (USF) [17], and Nuclear Factor-kappa B (NF-κB) [8, 16]. Mutagenesis of a conserved USF site has been shown to diminish the activity of the HIV-1 antisense promoter in reporter constructs [17]. The HIV-1 3′ LTR also contains several conserved NF-κB binding sites, which are known to drive HIV-1 antisense transcription. Point mutations in these sites have been demonstrated to down-regulate the activity of the antisense promoter in HIV-1 LTR reporter constructs [8, 15, 17]. NF-κB-activating agents, such as phorbol 12-myristate 13-acetate (PMA) and tumor necrosis factor α (TNFα), are also known to induce HIV-1 antisense transcription, likely through increased binding of NF-κB on the viral 3′ LTR [6, 8, 15]. In line with this finding, HIV-1 antisense LTR reporter plasmids containing mutated κB sites demonstrate decreased responsiveness towards PMA and TNFα stimulation [6, 8]. PMA stimulation of antisense transcription in a luciferase-expressing HIV-1 proviral DNA clone has also been demonstrated in both transfection and infection experiments [6]. In comparison, TNFα-mediated induction of HIV-1 antisense transcription remains debatable, as other studies have failed to replicate this phenomenon [6]. Thus, accumulating evidence illustrates an important role of NF-κB in the induction of HIV-1 antisense transcription. However, our bioinformatics analysis of the HIV-1 antisense promoter suggests many additional transcription factors likely contribute to the overall regulation of negative-sense transcripts in this retrovirus (Fig. 1, Table 1).

**Table 1:** Comparison of the types of cellular transcription factors and the number of their cognate binding sites on the antisense promoters of human-specific ERVK, HIV-1, and HTLV-1. Sequences of the consensus binding sites for cellular transcription factors predicted to bind these retroviral antisense LTRs are also shown

Transcription Factor	Consensus binding site sequence	Number of putative binding sites predicted in 3′ LTR		
		ERVK HS	HIV-1	HTLV-1
Activating Protein 1 (AP-1) c-Jun/c-Fos	TGA (G/C) TCA	5	3	2
Activating Protein 2α (AP-2α)	GCCNNNGGC	5	2	14
Activating Transcription Factor-3 (ATF-3)	T (T/G) ACGT (A/C) (A/G)	1	---	3
Androgen Receptor (AR)	GG (A/T) ACANNNTGTTCT (**ARE**)	1	---	---
Aryl hydrocarbon receptor / AhR nuclear translocator (AhR/ARNT)	TNGCGTG	2	3	4
cAMP Response Element Binding Protein (CREB)	TGACGTCA	---	---	2
CCAAT-Enhancer Binding Protein (C/EBP)	A (A/G/T) C (C/A) AAT	7	3	3
Cellular Myeloblastosis virus protein (c-Myb)	(T/C) AAC (G/T) G	12	7	8
Cellular Myelocytomatosis virus protein (c-Myc)	NNNCACGTGNN (**E-box**)	10	1	2
Chicken Ovalbumin Upstream Promoter Transcription Factor-1 (COUPT or COUPTF-1)	(A/G) G (G/T) TCA	7	15	13
E2 Factor-1 (E2F-1)	TTT (C/G) (C/G) CGC	---	---	2
Early B-cell Factor (EBF)	CCCNNGGG	9	4	7
EGFR-specific Transcription Factor (ETF)	G/C rich regions	14	4	19
Enkephalin Transcription Factor-1 (ENKTF-1)	TGGCGTA	1	---	---
Estrogen Receptor (ER-α; ER-β)	GGTCANNNTGACC (**ERE**)	1	---	---
E-twenty six (ETS-1; ETS-2)	GGA (A/T)	12	8	7
ETS-like Transcription Factor 1 (Elk-1; ELF-1)	CAGGAA (C/G) (**PU Box**)	5	1	2
Forkhead Box P3 (FOXP3)	(G/A) (T/C) AAACA	14	1	3
GATA binding proteins GATA - 1, 4, 5 GATA - 2, 3 GATA - 6	(A/T) GATA (A/G) (**GATA Box**) (A/T) GAT (A/C) (A/G) (A/T) GAT (T/C) (A/G)	3	2	1
		6	6	2
		7	5	2
GC Binding Factor (GCF)	GCGGGGC	---	---	1
Glucocorticoid Receptor (GR-α; GR- β)	GGTACANNNTGTTC (**GRE**)	1	---	---
Hepatocyte Nuclear Factor-3/4 (HNF3; HNF4)	GGTCA repeats	3	---	3
Hepatocyte Nuclear Factor-1 (HNF1)	GTTAATNATTAAC	1	---	---
Hypoxia Inducible Factor-1 (HIF-1)	(A/G) CGTG (flanked by (A/C) ACAG)	3	1	2
Ikarose-1 (IK-1)	TGGGA (A/T)	16	5	6
Interferon Regulatory Factors (IRF)	GAAANN repeats (**ISRE**)	2	---	2
Lymphoid Enhancer-binding Factor 1 (LEF-1)	CTTTGAA	3	---	---
Myc Associated Zinc finger protein (MAZ)	GGGAGGG	9	1	6
Nuclear Factor of Activated T cells (NFAT)	GGAGAA	10	---	7
Nuclear Factor I (NFI; NFI/CTF)	TTGGCNNNNNGCCAA	---	---	2
Nuclear Factor Kappa B (NF-κB)	GG (G/A) (G/A) NN (C/T) (C/T) CC	9	7	6
Nuclear Factor-Y (NF-Y)	CCAAT	2	---	3
Polyomavirus Enhancer Activator 3 (PEA or PEA-3)	GGA (A/T)	12	8	7
Progesterone Receptor (PR-A; PR-B)	GNACANNNTGTNC (**PRE**)	1	---	---
Protein 53 (p53)	CATTAG	8	1	1
Recombination signal Binding Protein-Jκ (RBP-Jκ)	(C/T) GTGGGAA	1	---	---
Retinoid X Receptorα (RXRa)	AGGTCA	11	4	7
Serum Response Factor (SRF)	C (C/T) (A/T)_6_GG	2	4	2
Sex-determining Region Y (SRY)	(A/T) (A/T) CAA (A/T)	12	4	3
Signal Transducers and Activators of Transcription (STAT)	TTCNNNNGAA	10	4	8
Specificity Protein-1 (Sp1)	GGGCGG	15	1	14
TATA Binding Protein (TBP)	TATAAA	1	---	1
T cell Factor-4E (TCF-4E)	(G/C) ATCAAAGG	1	---	---
Thyroid Hormone Receptor (TRβ)	TGAGGTCA (**TRE**)	1	---	1
Transcription Factor II-D (TFII-D)	TATAAA	1	---	1
Transcription Factor II-I (TFII-I)	CANNTG	13	3	1
Upstream Transcription Factor (USF-1, USF-2)	CACGTG (**E-box**)	10	1	2
Vitamin D Receptor (VDR)	G (G/T) TCA	25	20	21
X-box binding protein (XBP-1)	CCACG	9	9	9
Yin Yang 1 (YY1)	GCCATNTT	1	---	1

--- indicates no putative binding sites

*ARE* androgen response element, *E-box* enhancer box, *ERE* estrogen response element, *PU Box* purine box, *GRE* glucocorticoid response element, *ISRE* interferon stimulated response element, *PRE* progesterone response element, *TRE* thyroid hormone response element

In addition, there is evidence of single nucleotide polymorphisms in transcription factor binding sites within the LTRs of different HIV-1 subtypes [18–21]. This sequence variation leads to subtype-specific differences in proviral gene expression, thereby imparting unique biological characteristics to a given strain. This is well illustrated for the 5′ LTR of HIV-1 subtype E, which harbors a shift from an NF-κB to a GABP binding site [20]. Abolished NF-κB binding to this LTR does not lead to a loss of promoter function *in vitro*; instead, gain of GABP binding to this mutated NF-κB site enhances Tat-mediated HIV-1 gene expression in several cell types, as well as improves virus replication in SuPT1 cell line [20]. Thus, variability in transcription factor binding sites in retroviral LTRs can have a positive impact on proviral gene expression under certain conditions – this may serve to enhance retroviral fitness and spread. Likewise, subtype-specific variations in the HIV-1 3′ LTR may also exert another layer of control over the proviral antisense transcription.

Further, retroviral proteins can also modulate proviral antisense transcription. Conflicting results have been reported on the potential role of the HIV-1 accessory protein Tat in regulating antisense transcription. Tat has been reported to enhance HIV-1 antisense transcription in cell lines co-transfected with a Tat expression vector and luciferase reporter plasmids containing HIV-1 3′ LTR but not 5′ LTR [6]. However, in studies utilizing luciferase reporter constructs containing both HIV-1 3′ LTR and 5′ LTR, Tat was not shown to alter antisense transcription [22, 23]. Likewise, in a separate study utilizing HIV-1 3′ LTR luciferase reporter plasmids, overexpression of Tat did not influence antisense luciferase activity [8]. Thus, the role of Tat in regulating HIV-1 antisense transcription remains controversial and should be confirmed in the context of full length proviruses integrated during HIV-1 infection, rather than in artificial reporter assays. As there is no evidence of TAR RNA synthesis during antisense transcription, the mechanism by which Tat influences the activity of the HIV-1 antisense promoter remains unknown. It is possible that the interaction of Tat with cellular transcription factors, such as Sp1, modulates their binding to the HIV-1 3′ LTR [6], which may affect the extent of HIV-1 antisense transcription.

### HTLV Antisense transcription

A large portion of our knowledge on retroviral antisense transcription stems from studies of Human T-Lymphotropic Viruses (HTLV), particularly HTLV-1. The HTLV-1 antisense genomic strand encodes a basic leucine zipper (bZIP)-containing protein, designated HBZ. Although HTLV-1 is capable of infecting different cell types *in vitro*, HBZ protein is mainly detected in CD4^+^ T cells *in vivo* [3, 24, 25]. This cell-type specific expression of HBZ has been shown to play a variety of roles in the pathogenesis of HTLV-mediated T-cell leukemia (reviewed in [3, 26]). For instance, HBZ transforms T-cells into a cancerous phenotype, in part by enhancing the expression of chemokine receptor CCR4 in this cell type, which promotes T-cell proliferation and migration [27]. HBZ also inhibits HTLV-1 sense transcription by recruiting essential transcription factors, such as CREB, away from the proviral sense promoter – this process facilitates HTLV-1 latency in infected T cells [3]. HBZ also affects many other cellular processes, including host gene expression, innate immune signaling, apoptosis, autophagy, and DNA repair – all of which further influence the pathology of the HTLV-1 infection (reviewed in [3]). Similar to HTLV-1, HTLV-2, HTLV-3, and HTLV-4 are equally capable of producing antisense proteins – APH-2, APH-3, and APH-4, respectively – though their functions have not been clearly elucidated [4, 26, 28–30].

**Fig. 2:**
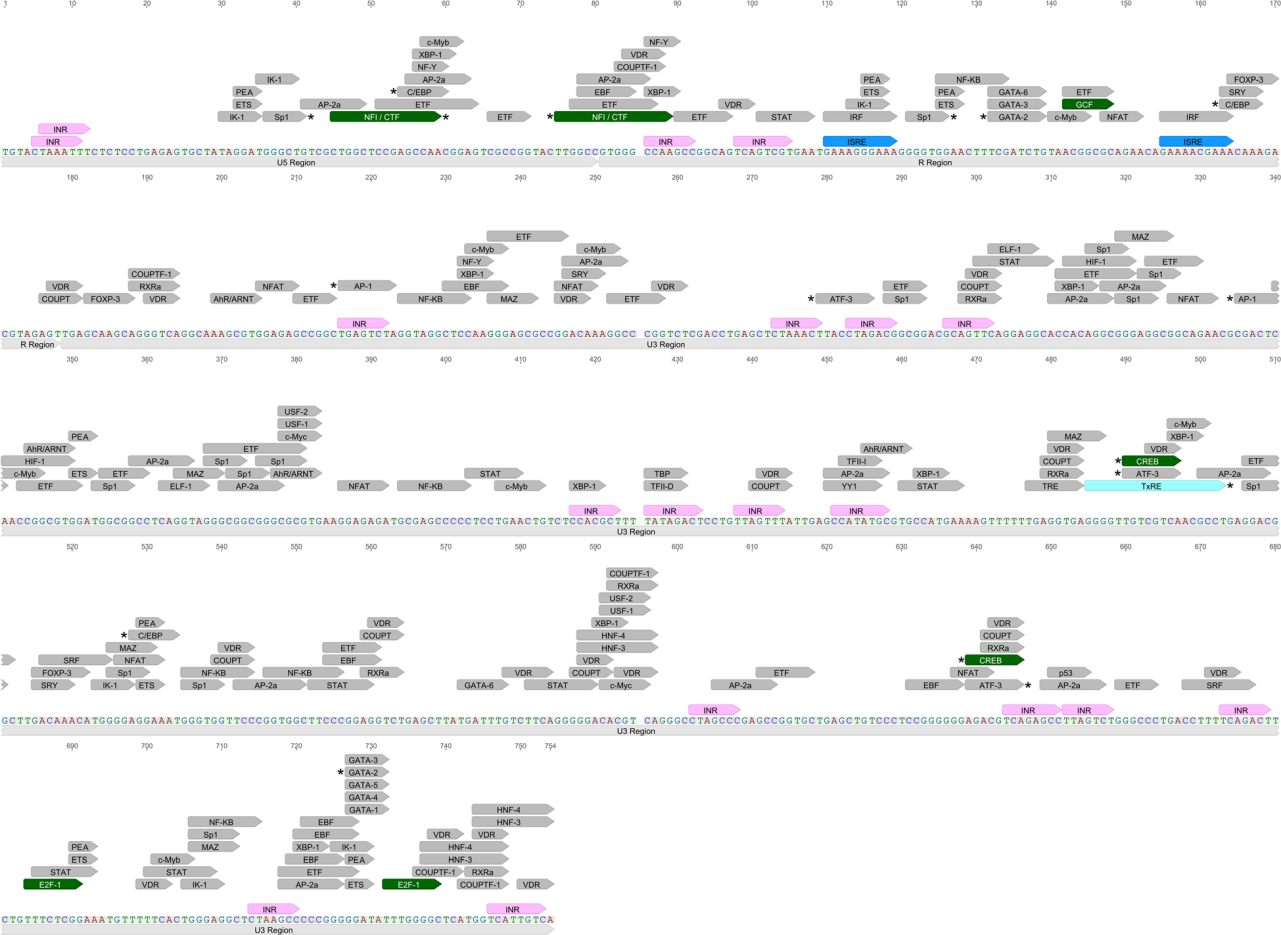
*In silico* examination of transcription factor binding sites and response elements within the representative HTLV-1 3′ LTR using ALGGEN-PROMO software [73]. The prototypic HTLV-1 3′ LTR sequence used was obtained from GenBank, accession number AB513134 (B1033-2009 isolate). The known binding sites for AP-1, ATF, C/EBP, CREB, GATA-2, NFI, and Sp1 in the HTLV-1 3′ LTR are flagged with an asterisk. Transcription factor binding sites unique to HTLV-1 are indicated in green. Initiator motifs (INRs) are indicated in pink. ISRE sites are indicated in blue. Abbreviations used include: INR = initiator motif, ISRE = interferon stimulated response element, and TRE = thyroid hormone response element, and TxRE = tax responsive element. Sequence annotations were performed using Geneious software [68]

Despite the extensive research focused on deciphering the role of HTLV-encoded antisense proteins in disease pathogenesis, there are a limited number of studies aimed at understanding the regulation of HTLV antisense transcription at the level of the proviral 3′ LTR. It has been demonstrated that HTLV-1 *hbz* is transcribed starting from the 3′ LTR of the HTLV-1 provirus [31–33]. Initiation of transcription is possible at several different positions within the R and U5 regions of the 3′ LTR [31]. Like HIV-1 antisense promoter, the HTLV-1 3′ LTR is a TATA-less promoter harboring many INR motifs, thus leading to a multitude of antisense transcription initiation sites [31, 34, 35]. The transcription of *hbz* relies heavily on three Sp1 sites in the U5 region of the proviral 3′ LTR [23, 31, 34, 36]. In luciferase assays, HTLV-1 antisense promoter activity is markedly reduced upon mutation of single or multiple Sp1 sites [34]. The same study identified binding sites for GATA binding protein-2 (GATA-2), cAMP responsive element binding protein (CREB), activating protein 1 (AP-1), and nuclear factor-1 (NF-1) in the HTLV-1 3′ LTR. However, mutations of each of these sites only reduced promoter activity slightly in luciferase assays [34]. Other cellular transcription factors, including activating transcription factor (ATF), CCAAT-enhancer binding protein (C/EBP), and histone acetyltransferase p300 have also been shown to bind the HTLV-1 antisense promoter in HTLV-1 transformed cell lines, as well as in cells derived from patients with Adult T-cell Leukemia/Lymphoma (ATL) [3, 37]. Thus, these transcription factors are postulated to play a role in regulating antisense HTLV-1 transcription. Whether they promote or inhibit antisense gene expression remains to be elucidated. In addition, T-cell factor 1 (TCF-1) and Lymphoid enhancing factor 1 (LEF-1) have been shown to slightly enhance *hbz* transcription and HTLV-1 3′ LTR activation in luciferase assays [38]. In line with these studies, bioinformatics analysis of the consensus HTLV-1 3′ LTR has not only confirmed the presence of intact binding sites for the aforementioned transcription factors, but has also revealed putative sites for numerous other antisense transcriptional regulators (Fig. 2, Table 1). Interestingly, some of the identified binding sites for transcription factors, notably ATF, CREB, and NF-I, are unique to the 3′ LTR of HTLV-1, and are not predicted within the HIV-1 3′ LTR. Thus, HTLV-1 antisense gene expression is likely regulated by a multitude of cellular and retroviral transcription factors. There is an evident need for future research characterizing the transcriptional regulators that broadly and selectively modulate antisense gene expression in the various tissue types targeted by retroviruses.

The transcription of HTLV-1 proviruses is further modulated by the antisense-encoded HBZ protein. HBZ binding to the 5´ LTR of HTLV-1 promotes viral latency by suppressing sense transcription [39]. Conversely, HTLV-1 antisense transcription is positively regulated by HBZ. HBZ has the capacity to form heterodimers with a cellular transcription factor JunD [36]. Co-expression of JunD and HBZ has been shown to significantly increase HTLV-1 3′ LTR activity in luciferase assays as compared to the expression of JunD or HBZ alone [36]. Also, luciferase activity was not enhanced with HBZ overexpression in knockout cells lacking JunD [36]. It was further shown that HBZ/JunD dimers are recruited to Sp1-bound regions of the HTLV-1 3′ LTR, due to the interaction of JunD with Sp1 [36]. Accordingly, mutation of one of these Sp1 sites in the HTLV-1 reporter construct, or the overexpression of Sp1 mutants lacking DNA-binding ability, resulted in a significant decrease in luciferase expression [36]. Therefore, HTLV-1 antisense transcription is regulated through interactions between HBZ, JunD, and Sp1 at the 3′ LTR.

As suggested for HIV-1 Tat interaction with its 3´ LTR, the HTLV-1 accessory protein Tax can also up-regulate the proviral antisense transcription. Overexpression of Tax has been shown to markedly enhance luciferase activity from transiently expressed, as well as stably integrated, HTLV-1 3′ LTR reporter constructs in human cell lines [40]. Tax responsive elements (TxREs) containing near-consensus CREB binding sites have been reported in the HTLV-1 antisense promoter [3, 34, 40]. Mutations of these TxREs, which render them incapable of interacting with CREB, exhibited a dramatically reduced luciferase activity from the 3′ LTR in the presence of Tax [34, 40]. Thus, viral Tax protein has been shown to drive HTLV-1 antisense transcription by cooperating with CREB at TxREs at the 3′ LTR. In stark contrast, several reports using similar methodology, but different host cells, have not detected Tax-mediated regulation of viral antisense transcription [23, 32, 41]. Thus, the discrepancies between these studies suggests that Tax-mediated regulation of antisense gene expression likely depends on the cell type being investigated, and consequently, the availability of cell-specific transcription factor complexes required for this process. This would be consistent with similar cell-type specific observations surrounding Tax-dependent transactivation of HTLV-1 sense transcription [41].

## Antisense transcription among other exogenous retroviruses

Antisense transcription is not exclusive to HIV and HTLV, and has also been reported in the deltaretroviruses bovine leukemia virus (BLV) [42] and simian T-cell leukemia virus (STLV) [43], the lentiviruses feline immunodeficiency virus (FIV) [44] and bovine immunodeficiency virus (BIV) [45], as well as the gammaretrovirus murine leukemia virus (MLV) [46]. However, the regulation of antisense transcription remains poorly studied in these retroviruses. A recent report has demonstrated that the antisense transcription of BLV, a close relative of HTLV-1, is regulated by an Interferon Regulatory Factor (IRF) binding site and an E-box in its 3′ LTR [42]. Through BLV 3′ LTR luciferase reporter assays, mutation of this IRF binding site or the E-box resulted in modest to significant downregulation of antisense luciferase activity, respectively. Bioinformatics analysis has revealed the presence of two putative intact IRF binding sites in the HTLV-1, but not HIV-1, representative 3′ LTR, as well the presence of intact E-boxes in both antisense promoters (Fig. 2, Table 1). This suggests that IRF may regulate the antisense transcription of select retroviruses, whereas E-boxes may be a broader feature of retroviral 3′ LTRs.

## Antisense transcription among endogenous retroviruses

Antisense transcription has been emerging as a common, but generally underappreciated, feature of ERV gene expression patterns. Several human ERVs, particularly ERV9 and ERVK loci, exhibit transcription from the antisense strand. Above and beyond the potential of antisense products to modulate endogenous retrovirus expression patterns, the impact of antisense viral products on human biology is becoming apparent. Most notably, antisense transcription of ERVs may play important roles in the regulation of human gene expression or modulation of cellular pathways.

### ERV9 antisense transcription

Among human endogenous retroviruses, antisense transcriptional regulation of ERV9 loci is the best understood. Several cellular transcription factors are known to induce the expression of antisense RNA from the U3 region (referred to as the U3 AS RNA) of the ERV9 LTR. This includes CREB, glucocorticoid receptors (GR), IRF, signal transducers and activators of transcription (STAT), and activating protein 2 (AP-2) [47]. Interestingly, the AUUGG motifs within the ERV9 antisense transcripts have been experimentally demonstrated to interact with and sequester select cellular transcription factors – NF-Y, p53 and Sp1 [47]. We have predicted the presence of similar motifs in the antisense RNA originating from the ERVK 3’ LTR (data not shown). By sequestering the aforementioned cellular transcription factors, ERV9 U3 AS RNA serves to repress the expression of genes involved in cell cycle activation, thereby inhibiting uncontrolled cellular proliferation. Accordingly, deregulation of this ERV-derived antisense RNA has the potential to promote tumor formation and propagation [47]. Thus, the production of endogenous AS RNA decoys may be an important phenomenon among endogenous retroviruses, and may serve essential regulatory and protective functions for their human hosts.

### ERVK antisense transcription

The human genome is ubiquitously populated with ERVK sequences including solitary LTRs and partial proviral sequences, as well as full-length proviruses. Solitary LTRs are the most abundant ERVK elements within the human genome, and are estimated to number over 25,000 [48]. They are frequently present in close proximity to our genes, and therefore may be involved in the regulation of neighbouring genes by acting as promoters or enhancers. It is estimated that at least 50% of human-specific ERVK (HML-2) LTRs serve as promoters for the transcription of human genes [49]. ERVK LTRs have been experimentally shown to activate the expression of promoter-less reporter genes in luciferase assays when inserted in both forward and reverse orientations, indicating their bidirectional promoter activity [48]. Such bidirectional activity lends plausibility to antisense viral RNA transcription mediated by the 3′ LTR of ERVK.

**Fig. 3:**
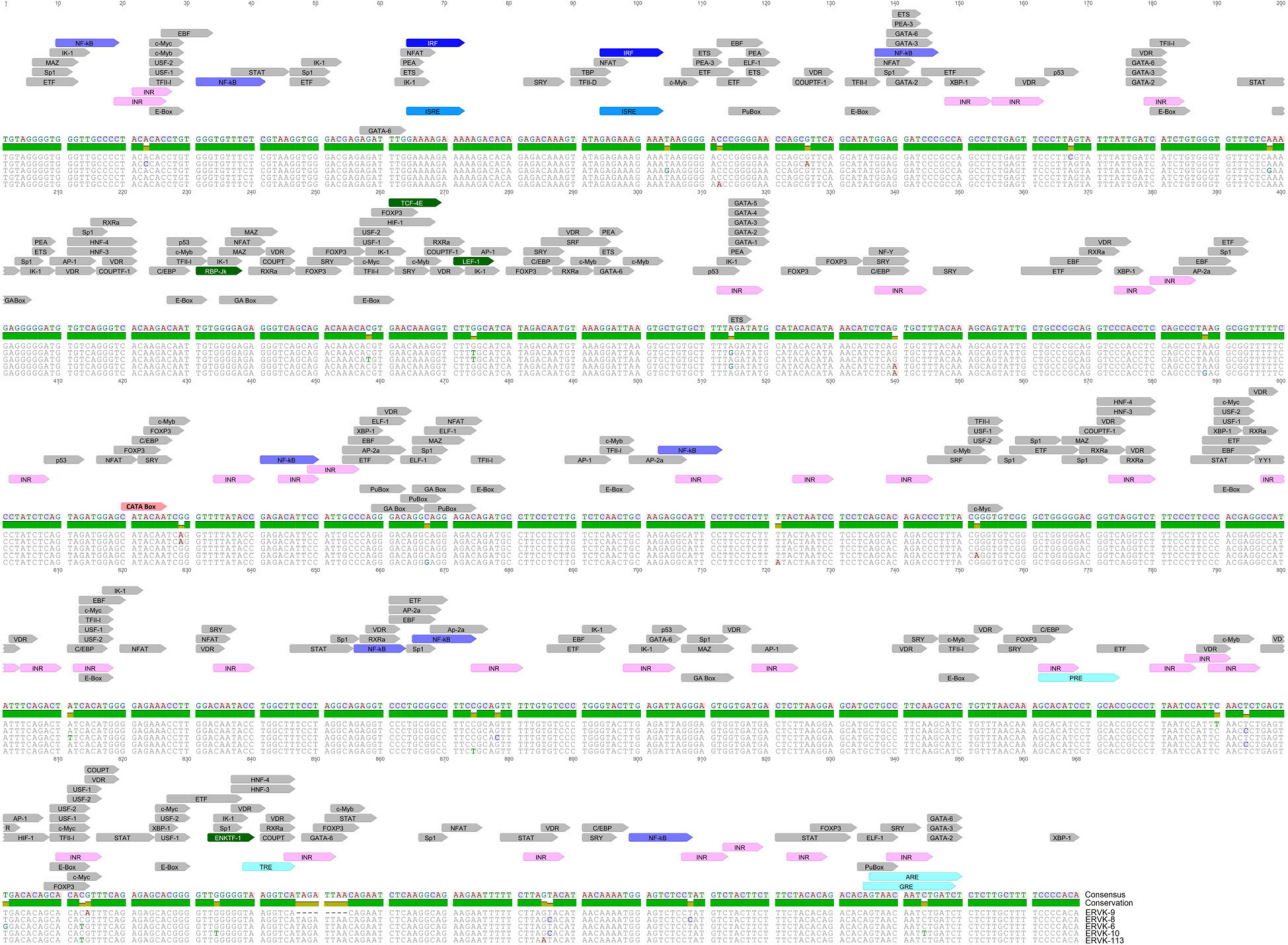
*In silico* examination of the conserved transcription factor binding sites and response elements within prototypic human-specific endogenous retrovirus-K (ERVK) 3′ LTRs using ALGGEN-PROMO software [73]. The ERVK 3′ LTR consensus sequence was constructed using individual ERVK LTRs in the following order (GenBank accession numbers in brackets): ERVK-9 (AF164615.1), ERVK-8 (AY0378929.1), ERVK-6 (AF164614.1), ERVK-10 (M12854.1), and ERVK-113 (AY037928.1). Conserved transcription factor binding sites are shown on the consensus sequence of the ERVK 3′ LTR. Unique transcription factor binding sites within the consensus ERVK 3′ LTR sequence are annotated in green. Initiator motifs (INRs) are indicated in pink. IRF and κB sites are indicated in dark and light purple, respectively. Hormone responsive elements are labeled in cyan. Abbreviations used include: ARE = androgen response element, E-box = enhancer box, ERE = estrogen response element, GRE = glucocorticoid response element, INR = initiator motif, ISRE = interferon-stimulated response element, PRE = progesterone response element, PU box = purine box, and TRE = thyroid hormone response element. Sequence alignment and annotations were performed using Geneious software [68]

Recently, several ERVK loci present outside human intronic regions have been demonstrated to exhibit transcription of the proviral antisense strand in prostate cancer cell lines. These include ERVK(I), ERVK-106, an un-named ERVK within locus 7q34, and multiple loci of solo LTRs [50]. When inserted in an opposite transcriptional orientation to that of their host intron, antisense transcription of ERVK proviruses can be explained as a consequence of host gene transcription. In contrast, the basis of transcription of the antisense strands of ERVK loci, such as ERVK-106, situated outside of human genes remains unclear. Though the regulation of antisense transcription driven by the ERVK 3′ LTR is poorly understood, it is likely mediated by a complex of TFs binding to the 3′ LTR (Fig. 3).

As a first step to better understand putative antisense ERVK transcription from its 3′ LTR, we performed an extensive bioinformatics analysis of 92 full-length ERVK HML-2 sequences, and predicted intact and conserved binding sites for numerous human transcription factors within 3′ LTRs of human-specific ERVK HML-2 proviruses (Fig. 3 shows transcription factor binding sites on five prototypic ERVK 3′ LTRs, and Table 1). Similar to antisense promoters of other retroviruses, conserved signatures of ERVK 3′ LTR include absence of a TATA-box and the presence of multiple conserved INR motifs scattered throughout the LTR (Fig. 3). This suggests that these putative alternative core promoter elements may initiate transcription from the ERVK proviral antisense strand at multiple sites. Additionally, in the absence of a TATA box (TATAAA), select subtypes of exogenous retroviruses, such as HIV-1 subtype E, have been shown to utilize a CATA box (CATAAA) to initiate proviral gene transcription [51]. Since the ERVK 3′ LTR contains a conserved putative CATA box (Fig. 3), ERVK may similarly use this promoter element to initiate antisense transcription.

It is noteworthy that we identified multiple Sp1 binding sites in the ERVK 3′ LTR, as they are critical for inducing transcription from TATA-less promoters. This is due to Sp1 recruitment of transcription factor II-D (TFII-D) which promotes the formation of transcription initiation complexes [16]. Since Sp1 binding to both HIV-1 and HTLV-1 antisense LTRs activates expression of their respective antisense proteins, this ubiquitous transcription factor may also have a key role in driving antisense transcription from the TATA-less ERVK 3′ LTR. Indeed, the ERVK 3′ LTR is laden with multiple conserved potential Sp1 binding sites; a total of 15 were identified, a similar density to that found of the HTLV-1 antisense promoter (Fig. 3). The ERVK antisense promoter harbors putative binding sites for other transcription factors known to induce the activity of 3′ LTRs of exogenous retroviruses and ERV9. These include docking sequences for STAT, AP-2, AP-1, USF, GATA, NF-Y, ATF, CBP, TCF, LEF, and E-box (Fig. 3).

In addition, the ERVK 3′ LTR contains multiple putative NF-κB binding sites (Fig. 3). Thus, this pro-inflammatory transcription factor may drive ERVK antisense transcription under select conditions, as documented for exogenous retroviruses [17, 26]. Another interesting feature of the ERVK 3′ LTR is the presence of two conserved consensus interferon stimulated response elements (ISREs). We have recently demonstrated the ability of IRF1 and NF-κB p65/p50 to synergistically enhance transcription from the ERVK sense promoter in the presence of select pro-inflammatory cytokines [52, 53]. Of note, ERVK 3′ LTR ISRE sequences are more similar to canonical ISREs as compared to their 5′ LTR counterparts, suggesting stronger IRF/NF-κB binding potential [54, 55]. Thus, inflammatory stimuli that enhance the activity of IRFs and NF-κB have the potential to provide an additional level of regulation on the ERVK antisense transcriptome.

Bioinformatics analysis further revealed select transcription factor binding profiles unique to ERVK antisense promoters, notably the presence of hormone responsive elements that were absent from both HIV-1 and HTLV-1 3′ LTRs. This includes the presence of putative binding sites for androgen (AR), estrogen (ER), glucocorticoid (GR), and progesterone (PR) receptors (Fig. 3, Table 1). Since these hormonal receptors are known to drive the activity of the ERVK 5′ LTR [53, 56, 57], they may also modulate the activity of its 3′ LTR.

The TF profile of the ERVK 3′ LTR may also point to cell-specific activation of antisense transcription. As with the HTLV-1 3′ LTR, the ERVK 3′ LTR contains putative binding sites for FOXP3, a transcription factor specific to regulatory T cells. Expression of antisense HTLV-1 transcripts in T regulatory cells is associated with the development of Adult T-cell Leukemia (ATL) [3, 24], potentially suggesting a shared mechanism for ERVK-associated leukemia [58]. In addition, several GATA family transcription factor binding sites are found within the ERVK antisense LTR. Owing to the importance of GATA family transcription factors in regulating immune cells [59], our data indicate that antisense ERVK expression may be modulated in hematopoietic cells, Moreover, impairment of GATA transcription factors are a hallmark of many cancers [60]. If ERVK were to employ an antisense product whose expression was i) driven by GATA proteins, and ii) held a similar latency-inducing function as HTLV-1 HBZ, the lack of GATA protein expression in cancers could explain the enhanced expression of sense-encoded ERVK protein products in transformed cells [58, 61].

Understanding the activity of the ERVK 3′ LTR promoter may be the key to elucidating the basis of antisense transcription of endogenous proviruses; however, it should be noted that not all ERVK LTRs are equally intact. Evaluation of human-specific and older 3′ ERVK LTRs in the HML-2 family reveals conserved, alternative and unique TF binding site profiles, when comparing recent and older provirus LTRs (data not shown). Therefore, developing an understanding of ERVK antisense transcription, especially in the context of specific genomic loci, is an area of research that clearly requires more investigation.

## ERVK genome harbors ORFs for putative antisense proteins

Since the ERVK antisense promoter contains conserved putative enhancer elements and consensus binding sites for numerous human transcription factors, it puts forth the question as to whether the ERVK antisense genomic strand contains open reading frames (ORFs) for putative antisense proteins. We have been able to identify conserved regions of ERVK antisense genome that resemble motifs found within glycosyltransferases (GTs) and thioredoxin/thioredoxin reductase (TRX) complexes (data not shown). Interestingly, one of these conserved motifs is located in a region complementary to the sense strand of ERVK env – a position similar to that of the open reading frames for *hbz* in HTLV-1, *aph-2* in HTLV-2, and *asp* in HIV-1 [9]. Interestingly, the production of viral-derived GTs or TRXs would be consistent with the needs of viruses [62], and more specifically retroviruses [63–65].

However, due to several limitations, it is currently difficult to predict with confidence whether the ERVK genome encodes antisense products. Notably, the primary structures of GTs and TRXs are extremely diverse and lack signature features [66, 67]. This lack of specificity in the predicted protein domains creates further issue for sequence alignment and does not lend assurance to current predictions without further bioinformatic and experimental investigation. It would further be worthwhile to employ whole transcriptome sequencing to examine the production of ERVK antisense transcripts in tissue specimens from patients with ERVK-associated diseases versus healthy controls. In the future, techniques developed to study antisense transcription in exogenous retroviruses will be useful in characterizing the expression of ERV antisense genomes.

## Conclusions

In the light of this report, further research on antisense transcription in endogenous retroviruses is warranted. We have shown that the exogenous and endogenous antisense LTRs share many regulatory similarities. Thus, it would be interesting to examine whether regulatory and pathological processes associated with exogenous retroviral antisense transcription are also applicable to ERVs. The presence of potentially new antisense-encoded transcripts and proteins would provide a more complete understanding of the biology of endogenous retroviruses, such as ERVK, and their roles in health and disease. A reconsideration of the nature of exogenous, as well as endogenous, retroviral transcription is required for a better understanding of *Retroviridae* as a whole.

## Methodology

The sequences of antisense promoters (3′ LTR) of exogenous retroviruses (HIV-1 and HTLV-1) and endogenous retrovirus-K (HML2) were obtained from GenBank, and reverse complemented in Geneious [68]. For HIV-1 and HLTV-1 3′ LTRs, the prototypic sequences used were that of the HXB2 and B1033-2009 strains, respectively, as these are the most commonly used reference sequences for these exogenous retroviruses [69–71]. The 92 ERVK (HML2) 3′ LTRs analyzed were grouped into human-specific or old sequences [72]. These were aligned separately in Geneious and a consensus sequence was obtained for each of the two groups. The human specific ERVK 3′ LTRs were further refined into five prototypic sequences, as each of the remaining ERVK 3′ LTRs exhibited transcription factor binding site patterns similar to one of these prototypic antisense promoters. These prototypic LTRs were aligned in Geneious-R6® software (version 6.1.7), and a consensus sequence was obtained. The binding sites for human-specific transcription factors within consensus HIV-1, HTLV-1, and ERVK (HML2) 3′ LTR sequences were predicted through ALGGEN PROMO database, which uses version 8.3 of TRANSFAC [73]. PROMO can be accessed at http://alggen.lsi.upc.es/cgi-bin/promo_v3/promo/promoinit.cgi?dirDB=TF_8.3. The search parameters used were: factor’s species – *Homo sapiens*, and site’s species – *Homo sapiens*. Each binding site for a given transcription factor was compared to the sequence of its known consensus binding site (listed in Table 1). The consensus binding sites for transcription factors predicted to interact with these retroviral promoters have been previously described [53]. The consensus binding sites for ATF3, AhR/ARNT, COUPTF1, E2F1, ETF, ENKTF1, FOXP3, GATA family, GCF, HNF, HIF, NF-Y, RxRα, SRF, TCF-4E, and TRβ were obtained from [74–90]. Only those sites with a maximum of one base pair deviation from the consensus binding sequence (or two for large hormonal response elements) were annotated on the target retroviral 3′ LTR. All annotations were performed in Geneious-R6®.

## Abbreviations

ASP: Antisense protein
ATL: Adult T-cell leukemia
BIV: Bovine immunodeficiency virus
BLV: Bovine leukemia virus
ERV: Endogenous retrovirus
FIV: Feline immunodeficiency virus
GT: Glycosyltransferase
HBZ: HTLV-1 bZIP factor
HIV: Human immunodeficiency virus
HTLV: Human T-cell lymphotropic virus
INR: Initiator motif
ISRE: Interferon stimulated response element
LTR: Long terminal repeat
MLV: Murine leukemia virus
STLV: Simian T-cell leukemia virus
TNFα: Tumor necrosis factor α
TRX: Thioredoxin reductase
